# Structure-activity relationship of anticancer drug candidate quinones

**DOI:** 10.55730/1300-0527.3647

**Published:** 2023-12-08

**Authors:** Nadire ÖZENVER, Neslihan SÖNMEZ, Merve YÜZBAŞIOĞLU BARAN, Merve YÜZBAŞIOĞLU BARAN, Ayşe UZ, Lütfiye Ömür DEMİREZER

**Affiliations:** 1 Department of Pharmacognosy, Faculty of Pharmacy, Hacettepe University, Ankara, Turkiye; 2 Department of Pharmacognosy, Gülhane Faculty of Pharmacy, University of Health Sciences, Ankara, Turkiye

**Keywords:** Quinone, naphthoquinone, cancer, cytotoxicity, alkannin, juglone

## Abstract

Breast cancer is one of the most prevalent cancer types worldwide. Chemotherapy is a substantial approach in the management of breast cancer despite the occurrence of chemotherapy-associated side effects and the development of multidrug resistance in cancer cells. At this point, a variety of quinone derivatives may represent potential as possible anticancer drug candidates due to possessing structural similarity towards clinically used anticancer drugs like doxorubicin. Therefore, we investigated the cytotoxic effects of various quinone derivatives with structural diversity towards a variety of breast cancer cells. We further determined their toxicity in healthy cells to evaluate their drug capability potential. Eighteen quinone derivatives (arbutin, hydroquinone, alkannin, lapachol, lawsone, juglone, aloe-emodin, aloin, cascaroside A (8-O-*β*-D-glucoside of 10-C*-β*-D-glucosyl aloe-emodin anthrone), chrysophanol, chrysophanol-8-O-*β*-D-glucoside, emodin, emodin-8-O-*β*-D-glucoside, frangulin A (emodin-6-O-*a*-L-rhamnoside), physcion, rhein, sennoside A, sennoside B (sennoside A and sennoside B are stereoisomers and rhein-dianthrone diglycosides in which *β*-D-glucose units are bound to the OH groups of rhein anthrones at their 8^th^ positions) were tested on MCF-7, SK-BR-3, MDA-MB-468, and MDA-MB-231 breast cancer cells and on H9c2 healthy rat cardiac myoblast cells in terms of their cytotoxicity and toxicity, respectively. The resazurin reduction assay was used to determine the cytotoxicity. Among the tested compounds, two naphthoquinone derivatives alkannin and juglone exhibited remarkable cytotoxicity on breast cancer cells and exhibited alleviated toxicity profiles on healthy cells deserving further investigation as possible drug candidates against breast cancer. Structure-activity relationships of these compounds were also evaluated and discussed. Alkannin and juglone, which are naphthoquinone derivatives isolated from natural sources, may be promising agents in the development of drug-candidate molecules with increased efficacy and safety for breast cancer.

## 1. Introduction

According to the data gained from the World Health Organization (WHO), cancer as a substantial cause of death globally accounted for approximately 10 million deaths in 2020 1 [1]. In 2018, 18.1 million new cases were announced worldwide. The number of new cancer cases and the number of cancer-related deaths are expected to rise to 29.5 million and 16.4 million annually by 2040, respectively 2. WHO reported that lung, prostate, colorectal, stomach, and liver cancers are among the most common types of cancer in men, while documenting breast, colorectal, lung, cervical, and thyroid cancers as among the most prevalent cancer types in women. WHO further declared 2.3 million women diagnosed with breast cancer and 685,000 breast cancer-related deaths globally in 2020. Even more, the number of patients diagnosed with breast cancer in the past 5 years was so high as of the end of 2020 that breast cancer was noted as the world’s most prevalent cancer type ^a^, 3, all emphasizing the importance of breast cancer globally. Although surgery, chemotherapy, radiotherapy, targeted therapy, immunotherapy and hormone therapy are the main treatment approaches for breast cancer^4^, chemotherapy holds rather a crucial place in the management of breast cancer. The chemotherapy approaches vary based on assorted parameters that are critical and affect the prognosis and progression of the disease (e.g., the amount of estrogen and progesterone receptors, human epidermal growth factor type 2 receptors (HER2/neu) in patients with breast cancer). Since, breast cancers are generally grouped according to the presence or absence of these receptors. If there are more estrogen and/or progesterone receptors than those of normal ones in breast cancer cells, it is expressed as estrogen and/or progesterone receptor positive and constitutes the majority (70%) of patients diagnosed with breast cancer. If there is a higher than normal level of HER2/neu gene and/or protein in the cancer tissue, breast cancer is called HER2/neu positive (15%–20%). Another group called triple-negative breast cancer affects 15% of breast cancer patients and lacks estrogen, progesterone, and HER2 receptors ^d^. These factors decide the selection of correct chemotherapy applications during therapy. Adriamycin, cyclophosphamide, paclitaxel, docetaxel, and their assorted combinations are the most widely used chemotherapeutics in breast cancer therapy [2]. However; the common side effects of chemotherapy and the development of multidrug resistance (MDR) against chemotherapeutics in cancer patients limit their clinical use [3]. MDR, a major obstacle in cancer therapy, causes the death of many patients. Therefore, new treatment regimens targeting MDR and their mechanisms of action are of prominence in cancer intervention.

Quinones possess a remarkable place among naturally-occurring secondary metabolites and they exhibit antitumor properties [4,5]. Doxorubicin is an anthracycline antibiotic widely used in the treatment of cancer in the clinic and has a 4-membered ring system containing chromophore, anthraquinone, and aminoglycoside [6–8]. It is considered one of the most effective cancer drugs available and is a first-line chemotherapeutic for breast cancer [9]. Despite the common uses of anthracycline antibiotics in the clinic, their serious side effects in healthy tissues and the development of drug resistance in cancer cells limit their clinical use. Cardiotoxic side effects and neurotoxicity are the most important side effects of anthracycline chemotherapeutics [10,11]. At this point, quinone derivatives may represent vital value in terms of their potential anticancer properties due to possessing structural similarity to the clinically used anthracycline antibiotics. Furthermore, quinones are secondary metabolites and exist in the composition of natural sources, which may make us consider the possibility that they may be relatively safe due to having a natural origin. To exemplify, structural relatives of naturally occurring quinones exist in different taxa of many plant families. These include Liliaceae (*Aloe* specs. etc.), Hypericaceae (*Hypericum* etc.), Polygonaceae (*Rheum*, *Rumex*, *Polygonum* specs. etc.), Rhamnaceae (*Rhamnus* specs. etc.), Rubiaceae (*Rubia*, *Galium* specs. etc.), Caeselpinaceae (*Cassia* spec. etc.), Boraginaceae (*Alkanna*, *Arnebia* spec. etc.), and Juglandaceae (*Juglans* spec., etc.) [12].

Nature offers great opportunities to obtain drug-candidate molecules and/or products with its extraordinary diversity in the prevention and/or treatment of diseases. In addition to traditional medicine applications, which is a treatment method dating back centuries, it forms a starting point in obtaining natural and (semi)synthetic drug molecules. Newman and Crag (2020) reviewed the existing data and reported that only 15.7% of the total number of small-molecule anticancer drugs approved between 1981 and 2019 were totally synthetic while others were associated with natural sources [13], all drawing a special emphasis on the naturally occurring agents.

In the present study, we investigated the cytotoxic effects of a number of quinone derivatives on a variety of breast cancer cells and their corresponding toxicities on healthy cells. Because, evaluating the literature data, we considered that they may become likelihood anticancer agents with high selectivity due to either being relatively safe compounds as they are of natural origin or presenting structural similarities towards clinically used anthracycline antibiotics. Furthermore, we assumed that their (semi)synthetic derivatives with enhancing cytotoxicity and fewer side effect profiles may be developed as potential drug leads in future studies.

Emerging scientific investigations have revealed their biological activities and therapeutic potential as drug-candidate quinones in preclinical and clinical investigations. Benzoquinone derivatives arbutin and hydroquinone, for instance, displayed assorted biological activities ranging from depigmentation to antitumor properties [14,15]. Hydroquinone, benzenediol containing benzene core possessing two hydroxy substituents para to each other [16], has been well-studied to date including a number of clinical studies. The current data has pointed out it has diverse activities with a special focus on depigmentation [17]. Arbutin, the beta-D-glucopyranoside of hydroquinone, is another benzoquinone derivative and has been displayed to possess various biological activities such as antitumoral, antimelanogenic, antiinflammatory and hepatoprotective, cardioprotective and antioxidant properties [18–21]. Evaluated the impacts of bicyclic naphthoquinone derivatives and their naturally occurring and/or synthetic derivatives, they were mostly known for their potent toxicities [22–25]. Alkannin, juglone, lapachol, and lawsone are hydroxy derivatives of 1,4-naphthoquinone skeleton differing in terms of the positions of their carrying-hydroxy and/or side chain groups that determine their activity profiles. Diverse activities including their antitumoral features along with the background mechanisms as well as their dyeing properties are well-studied in previous investigations [26–33]. When it comes to the tricyclic anthraquinone derivatives which comprise a huge part of our research, anthraquinone aglycones, their glycosides, and their dimers have been examined for their therapeutic potential to date. The well-known characteristic of 1,8-dihydroxyanthraquinone derivatives is their laxative properties [34]. Besides, their biological influences (with a special emphasis on cancer) of aloe-emodin [35], aloin [36], chrysophanol [37,38], emodin [39,40], physcion [41], rhein [42], sennoside A, and sennoside B [43,44] were revealed. Furthermore, emodin-8-O-*β*-D-glucoside and chrysophanol glycoside-containing extracts have been demonstrated to exhibit antitumoral properties, implying the probable cytotoxic action of their glycosidic derivatives [45,46].

**Figure 1:**
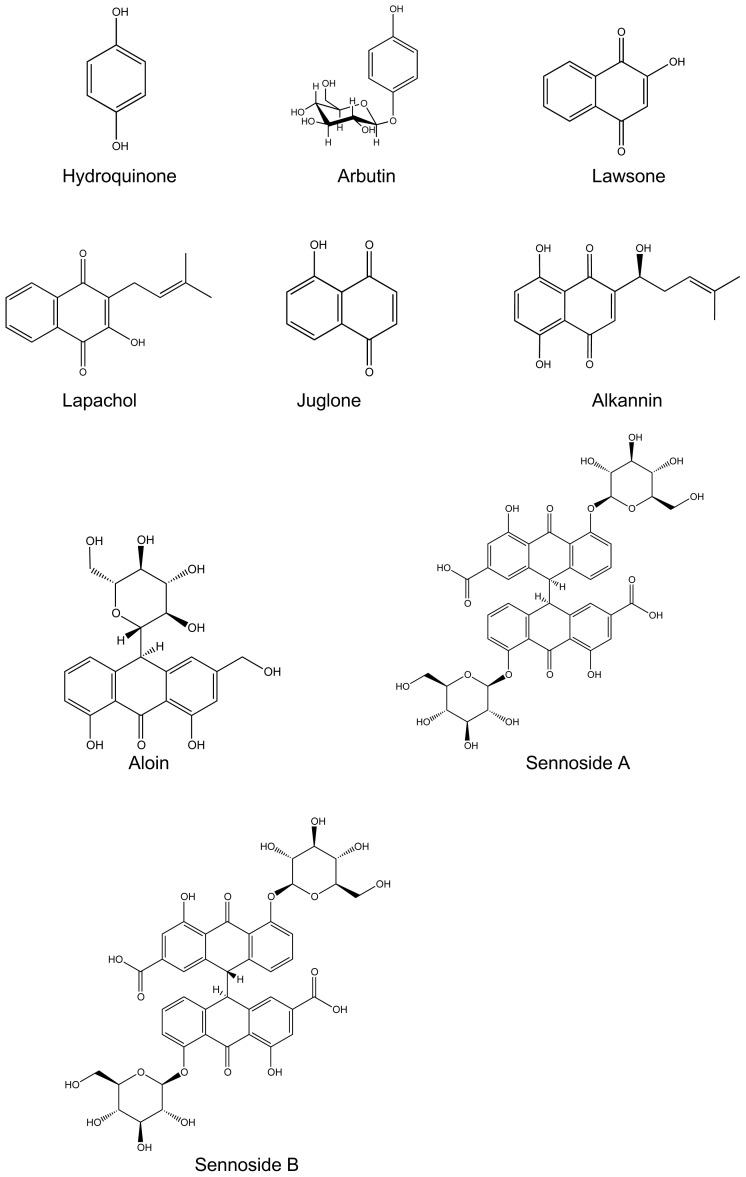

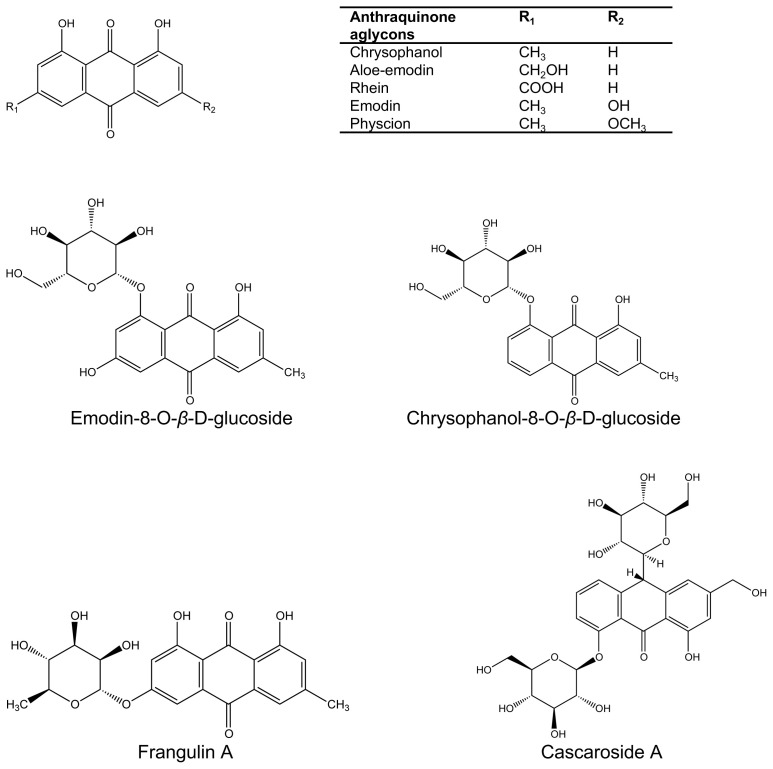
The chemical structures of the quinone derivatives investigated within the context of the present research.

Based on the accumulating evidence, we believe that quinone derivatives hold great importance in terms of their antitumor potential and require comprehensive monitoring. At this point, although assorted findings pointed out their probable action against a particular breast cancer cell line, the data usually was not enough to come to a general assumption. In the present research, we aimed to test various quinone molecules with structural diversity that occurs due to the oxidoreduction and dimerization reactions or the ring numbers (single, double, and triple) and sugar molecules in their composition, on breast cancer with different origins and thus to come through a broad assessment based on their specialized structures. Therefore, we examined the cytotoxicity profiles of 18 quinone derivatives (Figure 1) (arbutin, hydroquinone, alkannin, lapachol, lawsone, juglone, aloe-emodin, aloin, cascaroside A (8-O-*β*-D-glucoside of 10-C*-β*-D-glucosyl aloe-emodin anthrone), chrysophanol, chrysophanol-8-O-*β*-D-glucoside, emodin, emodin-8-O-*β*-D-glucoside, frangulin A (emodin-6-O-*a*-L-rhamnoside), physcion, rhein, sennoside A, sennoside B (sennoside A and sennoside B are stereoisomers and rhein-dianthrone diglycosides in which *β*-D-glucose units are bound to the OH groups of rhein anthrones at their 8^th^ positions) towards MCF-7, SK-BR-3, MDA-MB-468, and MDA-MB-231 breast cancer cell lines. Additionally, we demonstrated their acts on H9c2 rat cardiac myoblast cells for the determination of their possible cardiotoxicity and impact on healthy cells. Thus, we believe that our findings may form a basis for the discovery of potential therapeutic agents in the management of breast cancer.

## 2. Materials and methods

### 2.1. Chemicals

The tested compounds were purchased from commercial sources. Hydroquinone, aloin and emodin were purchased from Merck, Türkiye, while arbutin, lawsone, lapachol, juglone, chrysophanol, physcion, rhein, cascaroside A, chrysophanol-8-O*-*β-D-glucoside, frangulin A, sennoside A, sennoside B were commercially obtained from Sigma, Türkiye. Aloe-emodin, alkannin, and doxorubicin were provided from Cayman, Türkiye. On the other hand, emodin-8-O-β-D-glucoside was previously isolated from the roots of *Rumex acetosella* L. in our laboratory [35].

### 2.2. Cell culture

The cell lines used in the present work, their origins, and maintenance conditions were previously reported 5. The cell lines were obtained from the American Type Cell Culture (ATTC). The cell lines used for experimental studies were MCF-7 (HTB-22^TM^, ATTC), MDA-MB-231 (HTB-26^,^ ATTC), MDA-MB-468 (HTB-132, ATTC), SK-BR-3 (HTB-30, ATTC), and H9c2(2-1) (CRL-1446, ATTC). The origins of the cell lines depend on human breast epithelial (MCF-7, MDA-MB-231, MDA-MB-468, and SK-BR-3) and healthy rat myoblast (H9c2) cells.

### 2.3.Resazurin reduction assay

A resazurin reduction assay was conducted to test the cytotoxicity of the compounds. This assay is based on the reduction of resazurin to resorufin by viable cells [47]. Nonviable cells do not show a blue staining because they lost their metabolic capacity preventing resazurin reduction.

We preferred to apply the resazurin reduction method instead of other methods such as MTT or MTS assays which are also widely used assays. Several methods exist for determining cytotoxicity based on different mechanisms. The substantial reason why we chose the resazurin reduction assay is that it is a comparatively inexpensive and more sensitive method than those of tetrazolium assays such as MTT and MTS. Besides, it uses a homogeneous format and may be easily multiplexed with other assays, enabling the accumulation of further knowledge about the background mechanisms behind the cytotoxicity [48,49]. We also conducted a lot of investigations by using the resazurin reduction assay, confirming that it is among our areas of expertise [35,50,51].

Briefly, aliquots of 0.5 × 10^4^ adherent cells which were allowed to attach overnight, and 1×10^4^ suspension cells per well were seeded in 96-well-plates with or without the addition of varying concentrations of the test substance to get a total volume of 200 μL/well. After 72 h incubation and the addition of resazurin (Sigma-Aldrich) for 4 h, staining was measured by an Infinite 200 M Plex plate reader (Tecan, Türkiye) using an excitation wavelength of 544 nm and an emission wavelength of 590 nm. Each assay was independently performed for at least three times, with six parallel replicates each. The protocol has been recently reported [52]. Fifty percent inhibition concentrations (IC_50_) represent the drug concentrations required to inhibit 50% of cell proliferation, which were fitted with nonlinear regression using GraphPad Prism7.

## 3. Results and discussion

In the present study, quinone derivatives (benzo-, naphto-, anthra-) were investigated in terms of their cytotoxicity on a variety of breast cancer cells with different origins by the resazurin assay. The breast cancer cell lines studied were MCF-7, SK-BR-3, MDA-MB-468, and MDA-MB-231. Within the context of this research, we aim to examine the cytotoxic effects of a variety of quinone derivatives and, thus to come to a comprehensive conclusion about their cytotoxicity based on their specialized structures. Because, we consider that the responses of various breast cancer cells towards the studied quinones will be affected by their chemical diversity due to the oxidoreduction and dimerization reactions or the ring numbers (single, double, and triple) and sugar molecules in their composition.

As an initial step, we tested the compounds at 15 μM for preliminary evaluation. Because the National Cancer Institute (NCI) and existing literature usually assess a substance as cytotoxic if IC_50_ value of which is less than 10 μM [53–55]. To stay on the safe side, we selected 15 μM as a cut-off IC_50_ value for preliminary assessment. We, therefore, ranked the compounds as probable cytotoxic agents in case they caused 50% or less than 50% cell survival at 15 μM on the tested breast cancer cells.

According to the preliminary assessment of cell viability % of the tested compounds at 15 μM, IC_50_ values were ranged based on the breast cancer cell type and the structural diversity of those compounds. As a general assumption, specifically, naphthoquinone derivatives exhibited potent cytotoxicity towards breast cancer cells among the other quinone derivatives. A benzoquinone derivative hydroquinone, two naphthoquinone derivatives alkannin and juglone, and an anthraquinone derivative aloe-emodin displayed less than 50% cell viability at 15 μM on various breast cancer cells (Table).

**Figure 2:**
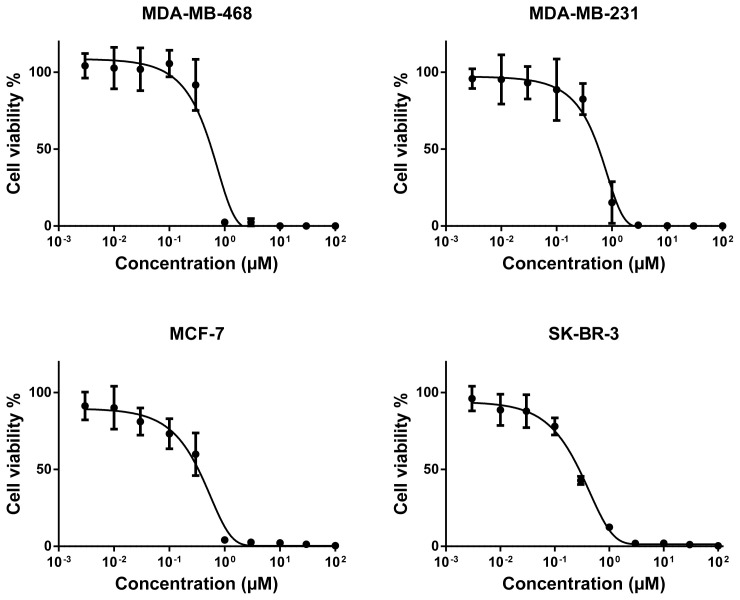
Cytotoxicity of alkannin towards MDA-MB-468, MDA-MB-231, MCF-7, and SK-BR-3 cell lines by means of resazurin assay. Mean values ± SD of three independent experiments are shown.

**Figure 3:**
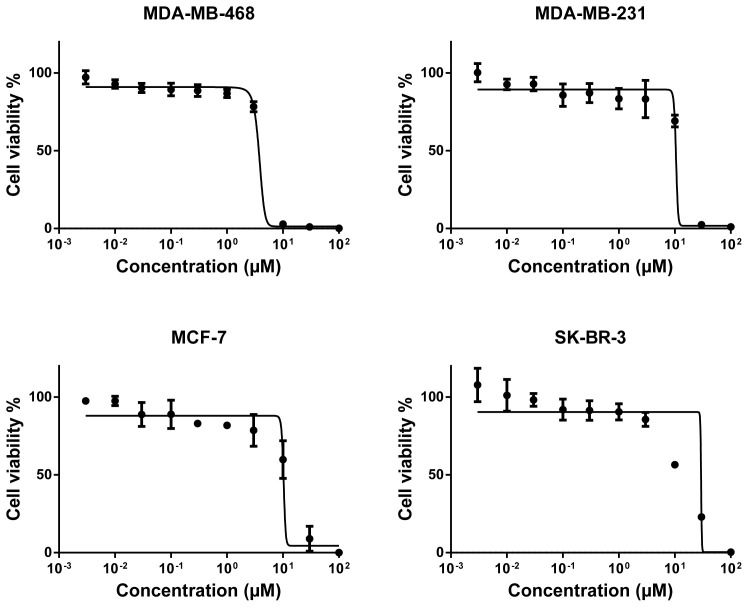
Cytotoxicity of juglone towards MDA-MB-468, MDA-MB-231, MCF-7, and SK-BR-3 cell lines by means of resazurin assay. Mean values ± SD of three independent experiments are shown.

**Figure 4:**
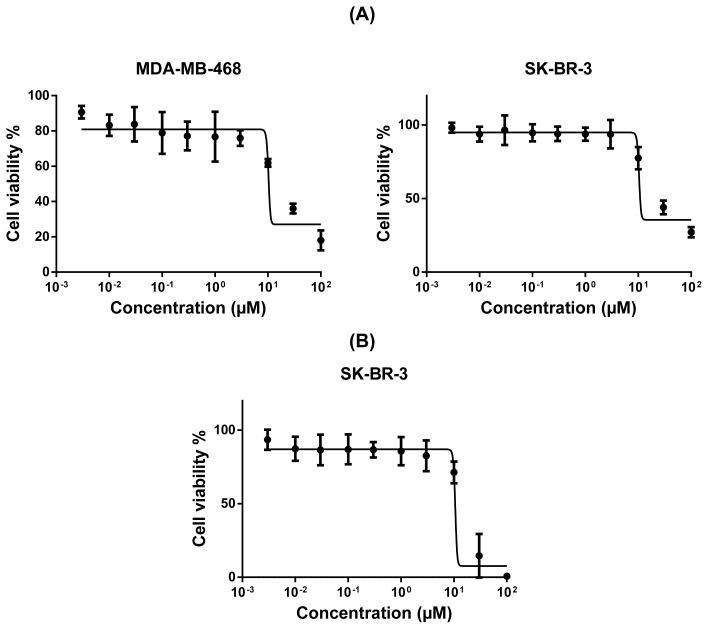
Cytotoxicity of aloe-emodin towards MDA-MB-468 and SK-BR-3 cell lines by means of resazurin assay (A). Cytotoxicity of hydroquinone towards SK-BR-3 cell lines by means of resazurin assay (B). Mean values ± SD of three independent experiments are shown.

Secondly, we further took those compounds into consideration for the determination of their dose-response curves at ranging concentrations and of their corresponding IC_50_ values and investigated their cytotoxicity in more detail on the breast cancer cells that they adversely affected as a result of preliminary evaluation. Four of these compounds (alkannin, juglone, hydroquinone, and aloe-emodin) were applied to the cell lines in which they were effectively cytotoxic at ranging concentrations (0.003–100 μM). Based on the dose-response curves of the compounds, the naphthoquinone derivative alkannin possessed the strongest cytotoxicity (Figure 2) followed by the other naphthoquinone derivative juglone (Figure 3) on all the tested breast cancer cells. Besides, the anthraquinone derivative aloe-emodin on both MDA-MB-468 and SK-BR-3 cell lines (Figure 4A) and the benzoquinone derivative hydroquinone on SK-BR-3 cells exhibited moderate cytotoxicity (Figure 4B).

The IC_50_ values of the compounds ranged from 0.26 μM to 26.4 μM for a variety of breast cancer cells. The IC_50_ values of the most cytotoxic compound alkannin towards MDA-MB-468, MDA-MB-231, MCF-7, and SK-BR-3 were 0.63 μM, 0.64 μM, 0.42 μM, and 0.26 μM, respectively. The IC_50_ values of juglone with the second highest cytotoxicity were 5.63 μM (MDA-MB-468), 15.75 μM (MDA-MB-231), 13.88 μM (MCF-7), and 13.89 μM (SK-BR-3). Furthermore, the IC_50_ value of the reduced benzoquinone derivative hydroquinone on SK-BR-3 was 17.5 μM while the corresponding IC_50_ values of anthraquinone aglycone aloe-emodin were 19.2 μM and 26.5 μM for MDA-MB-468 and SK-BR-3 breast cancer cells, respectively.

**Figure 5:**
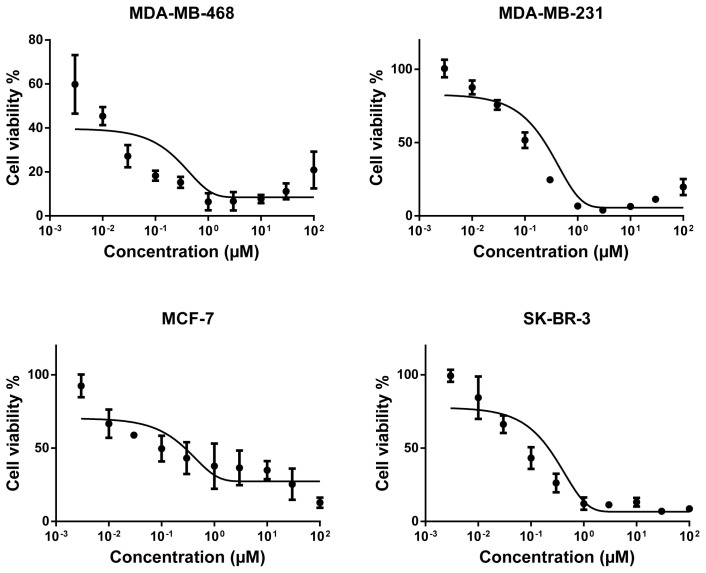
Cytotoxicity of doxorubicin towards MDA-MB-468, MDA-MB-231, MCF-7, and SK-BR-3 cell lines by means of resazurin assay. Mean values ± SD of three independent experiments are shown.

Clinically used doxorubicin was also tested on a variety of breast cancer cells as a positive control and potently repressed the cell viability on all the tested cell lines (Figure 5). The IC_50_ values of doxorubicin were 0.01 μM, 0.11 μM, 0.1 μM, and 0.08 μM for MDA-MB-468, MDA-MB-231, MCF-7, and SK-BR-3, respectively.

**Figure 6:**
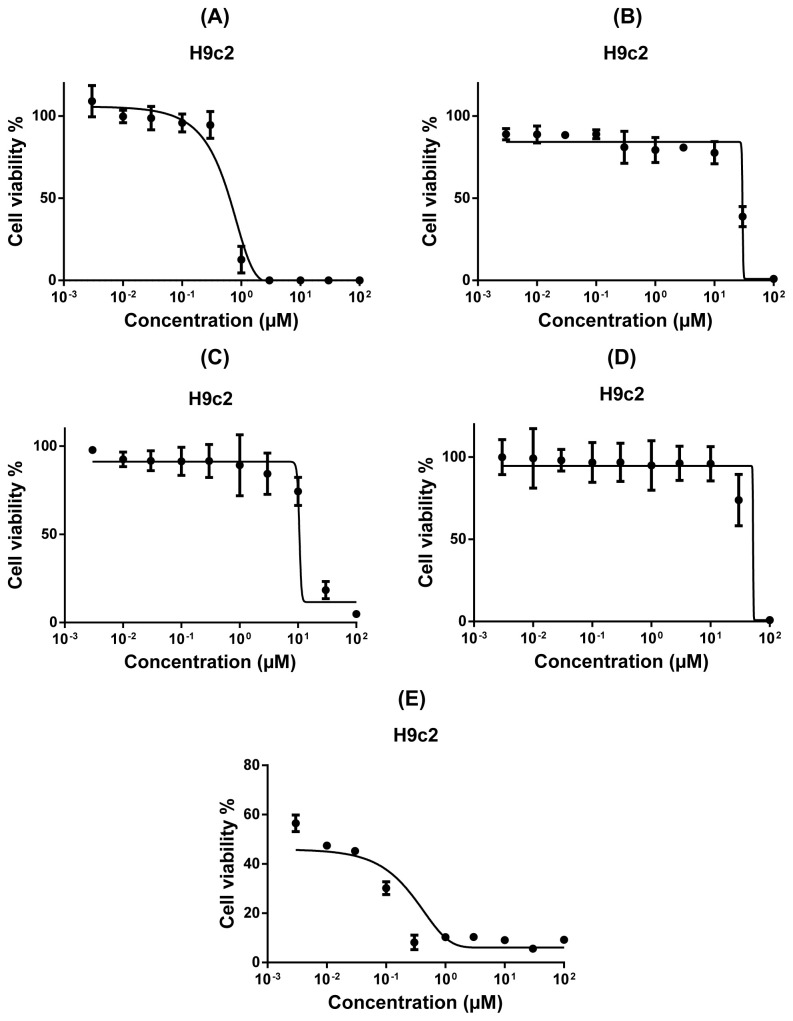
Toxicity of alkannin (A), juglone (B), aloe-emodin (C), hydroquinone (D), and doxorubicin (E) towards H9c2 rat cardiac myoblast cells by means of resazurin assay. Mean values ± SD of three independent experiments are shown.

Assessing the cytotoxic effects of these four quinone derivatives in more detail makes us think that the naphthoquinone derivatives alkannin and juglone may become promising compounds due to possessing low IC_50_ values while hydroquinone and aloe-emodin exhibited moderate cytotoxicity among the cell lines tested. Still, compared with the cytotoxicity of the tested compounds, the IC_50_ values of doxorubicin were quite low encountering the qualification of a chemotherapeutic agent. Despite huge differences between the IC_50_ values of doxorubicin and these four compounds with special emphasis on alkannin and juglone, we still insisted on considering if any of them may become a drug candidate not only in terms of their cytotoxicity but also due to their probable low toxicity. As cardiotoxicity is known to be one of the most common and serious side effects of anthracycline antibiotics including doxorubicin [56–58], we conceived that the cytotoxic compounds with low toxicity still hold promise as possible drug leads. Evaluating their toxicity on H9c2 rat cardiac myoblast cells, these four compounds were not toxic to the cells, contrary to doxorubicin which even killed half of the cells at the lowest concentrations it tested (0.003 μM) (Figure 6).

As shown in Figure 6E doxorubicin resulted in nearly 50% cell viability at the lowest concentration (0.003 μM) while the survival rates of H9c2 cells were much higher under the treatment of either alkannin or juglone than that of doxorubicin, suggesting that they hold therapeutic potential not only due to their cytotoxicity but also even more importantly because of their low toxicity profiles.

Chemotherapy is one of the substantial treatment options to cure breast cancer [59]. Despite many drugs or drug combinations approved by the FDA arecurrently in the clinic, their side effects still pose an incontrovertible issue affecting the quality of patients’ lives adversely [60–63]. At this point, the discovery of probable drug leads or drug combination regimens with fewer side effects will have great importance. As proof of that, in addition to the enhancement of cytotoxic activity, many studies have focused on looking for ways to relieve the undesirable side effect profiles [64–66]. Therefore, alkannin and juglone with high potency, require further investigation encountering relatively safe and potent cytotoxic compounds. Additionally, hydroquinone (on SK-BR-3) and aloe-emodin (on SK-BR-3 and MDA-MB-468) possessed moderate cytotoxicity while other quinone derivatives exhibit noncrucial cytotoxic effects.

Assessing the structure-activity relationships of the tested quinone derivatives in terms of their cytotoxicity on various breast cancer cells has drawn particular attention to naphthoquinone compounds. Two naphthoquinones alkannin and juglone possessed the strongest cytotoxicity among all the tested compounds, making us consider that these substances are the only compounds deserving further investigation due to possessing lower IC_50_ values than the cut-off value (15 μM). Ascertaining these 18 substances as a whole, our general assumption is that the naphthoquinone derivatives are of more importance than other tested benzoquinone and anthraquinone derivatives in terms of their cytotoxic properties. Comparing the cytotoxic activities of the tested naphthoquinones, we assume that the second, and the fifth positions of the *p*-naphthoquinone skeleton may hold importance in determining cytotoxic activity. The substitution on the 2nd position rather than the hydroxy group and the hydroxy groups on the 5th and the 8th positions of the 1,4-naphthoquinone skeleton may contribute to the emergence of potent cytotoxicity. Besides, comparing the structures of juglone and lawsone that differ only in terms of the location of a hydroxy group on the *p*-naphthoquinone skeleton, the movement of the hydroxy group from the 5th to the 2nd position of *p*-naphthoquinone led to the entire loss of cytotoxicity, remarking the significance of the 5th position. The comparison of alkannin and lapachol both of which contain alkyl substitution in their 2nd positions indicated that the nonsubstituted-3rd position and the presence of hydroxy groups in the 5th and the 8th positions may be required for a strong cytotoxicity profile. Before the determination of alkannin and juglone as promising agents with potent cytotoxicity, ongoing research was to ascertain their acts on healthy H9c2 cells. Toxicity findings were even rather meaningful, which verifies their safety. Because, unlike doxorubicin neither alkannin nor juglone exhibited potent toxicity profiles, implying their capacity as possible anticancer agents with alleviated adverse effects. Herein, the crucial term ‘selectivity’ has an undeniable prominence. Selectivity index (SI), calculated as the average of the IC_50_ value in healthy cells divided by the IC_50_ value of cancer cell lines, is an expression of drug capability, The higher SI value of a compound indicates the more selective property of that. An SI value greater than 2 suggests cytotoxic selectivity [67,68]. Assessed the SI values of two naphthoquinones with potent cytotoxicity, the SI value of juglone on MDA-MB-468 was 4.31 or came close to 2 for other tested cancer cell lines. For alkannin, the SI value was 2.62 for SK-BR-3 or above 1 for other cancer cells. On the other hand, the SI value of the clinically used drug doxorubicin was below 1 for MDA-MB-231, MCF-7, and SK-BR-3 and around 1 for MDA-MB-468. In this context, we believe juglone and alkannin along with their greater safety profile represent remarkable potential as a probable therapeutic agent against breast cancer for future studies.

Quinone derivatives are classified according to their ring structures such as 1,4-benzoquinone (cyclohexadienedione), 1,2-benzoquinone (ortho-quinone), 1,2-naphthoquinone, 1,4-naphthoquinone or 9,10-anthraquinone. The quinone moiety consists of an unsaturated benzene ring in which two oxygen atoms are bonded as carbonyl. The 1,4-benzoquinone portion of the molecule is a common structural feature in many compounds that received great interest due to their broad spectrum of biological activity [69]. Naphthoquinones, another important and common chemical class of the quinone family, may be observed in monomers, dimers, and trimers. The chemical structure of monomeric naphthoquinones depends on the bicyclic system. Those with substituted naphthalene skeletons in the C (1) and C (4) (1,4-naphthoquinones) or C (1) and C (2) (1,2-naphthoquinones) positions are the most common naphthoquinone groups in nature. The 1,4 (*para*)-naphthoquinones with the highest cytotoxic activity among the naphthoquinones contain two quinone groups that have the ability to accept one or two electrons to form radical anion or di-anion species. They show a strong cytotoxic effect, possibly due to the quinone redox cycle that causes the formation of reactive oxygen species (ROS) and the arylation reactions responsible for the biological effect in quinones [70]. In our previous study, alkannin and its derivatives obtained from *Arnebia densiflora* exhibited cytotoxic activity towards murine fibroblast (L929) cells, human laryngeal cancer (HEp-2) cells, and cervical cancer (HeLa) cells. The outcomes of DNA fragmentation and caspase-3 enzyme activity experiments in HeLa cell lines showed that these compounds exerted cytotoxic effects via apoptosis [71]. Anthracyclines are among the widely used antitumor agents [72] among which doxorubicin and daunorubicin are the leading drugs clinically used in the treatment of cancer [73,74]. Anthranoids have structural similarities to clinically used anthracycline antibiotics and can be used in the treatment of various malignancies. In our previous study, aloe-emodin was presumed as a promising anticancer drug candidate molecule due to inducing S and G2/M blockade during mitosis, disrupting mitochondrial membrane potential, causing the formation of ROS, as well as leading apoptosis and necrosis in T-lymphoblastic leukemia (CCRF-CEM) cells [35]. Existing data showed quinone derivatives as potential anticancer agents [75–78], which proved our findings in the present study.

## 4. Conclusion

As a consequence of the comprehensive examination of a variety of quinone derivatives on assorted breast cancer cells as well as healthy H9c2 rat cardiac myoblast cells, we suggested a broad evaluation of these compounds in terms of their cytotoxicity and toxicity. We believe that the chemical diversity of quinone derivatives will affect the responses of breast cancer cells and healthy cells. Inevitably, our expectations were verified by our findings on breast cancer cells. Among the 18 tested quinone derivates, specifically, alkannin and juglone were represented as promising drug leads that they both exhibited comparable cytotoxicity and greater safety than that of doxorubicin, suggesting those as potential anticancer agents deserving further investigations for the development of their semi(synthetic) derivatives with improved efficacy and safety.

## Figures and Tables

**Table:** Cytotoxic effect of quinone derivative compounds on a variety of breast cancer cell lines (MDA-MB-468, MDA-MB-231, MCF-7, SKBR-3) at 15 μM (n ≥ 3).

Benzoquinone derivatives (monocyclic quinones)	Cell survival (%) MDA-MB-468 cell line	Cell survival (%) MDA-MB-231 cell line	Cell survival (%) MCF-7 cell line	Cell survival (%) SK-BR-3 cell line
Arbutin	84.81 ± 8.39	89.57 ± 1.47	89.45 ± 12.27	104.90 ± 17.76
Hydroquinone	69.46 ± 7.34	96.39 ± 2.22	95.09 ± 15.72	**45.52 ± 0.89**

**Naphthoquinone derivatives (bicyclic quinones)**	**Cell survival (%)** **MDA-MB-468 cell line**	**Cell survival (%)** **MDA-MB-231 cell line**	**Cell survival (%)** **MCF-7 cell line**	**Cell survival (%)** **SK-BR-3 cell line**

Alkannin	**0.00**	**4.25 ± 5.45**	**1.31 ± 0.42**	**1.06 ± 0.53**
Lapachol	79.11 ± 2.43	75.86 ± 7.27	86.74 ± 10.75	91.20 ± 8.30
Lawsone	88.59 ± 4.38	99.66 ± 1.01	141.25 ± 27.53	139.42 ± 12.33
Juglone	**1.78 ± 1.53**	**1.99 ± 0.98**	**47.69 ± 4.81**	**24.48 ± 7.61**

**Anthraquinone derivatives (tricyclic quinones)**	**Cell survival (%)** **MDA-MB-468 cell line**	**Cell survival (%)** **MDA-MB-231 cell line**	**Cell survival (%)** **MCF-7 cell line**	**Cell survival (%)** **SK-BR-3 cell line**

Aloe-emodin	**41.78 ± 5.21**	64.08 ± 5.15	75.15 ± 1.59	**48.86 ± 3.13**
Aloin	87.80 ± 3.51	81.84 ± 4.24	80.16 ± 11.98	102.40 ± 5.59
Cascaroside A	85.50 ± 7.21	90.20 ± 7.13	79.33 ± 6.80	92.15 ± 9.00
Chrysophanol	80.67± 0.87	85.87 ± 7.52	87.40 ± 6.40	97.86 ± 6.89
Chrysophanol-8-O-*β*-D-glucoside	62.75 ± 1.72	84.02 ± 6.22	91.41 ± 1.73	65.93 ±16.46
Emodin	80.42 ± 8.98	66.35 ± 5.41	88.80 ± 0.99	64.84 ± 10.40
Emodin-8-O-*β*-D-glucoside	86.36 ± 0.87	102.93 ± 8.25	92.97 ± 9.25	90.52 ± 8.00
Frangulin A	51.25 ± 8.65	66.76 ± 1.90	77.34 ± 4.47	78.39 ± 4.35
Physcion	81.06 ± 6.95	77.60 ± 5.37	95.18 ± 10.23	91.11 ± 14.81
Rhein	82.06 ± 5.69	75.85 ± 7.83	95.39 ± 9.48	86.37 ± 2.68
Sennoside A	82.99 ± 4.18	87.99 ± 1.72	91.49 ± 6.61	90.77 ± 8.99
Sennoside B	85.87 ± 6.57	83.25 ± 9.62	82.69 ± 3.58	92.36 ± 10.04

## Footnotes

1 The data is obtained from WHO [accessed 14 02 2023]. (Website https://www.who.int/news-room/fact-sheets/detail/cancer)

2 The data is obtained from NCI [accessed 14 02 2023]. (Website https://www.cancer.gov/about-cancer/understanding/statistics)

3 The data is obtained from WHO [accessed 14 02 2023]. (Website https://www.who.int/news-room/fact-sheets/detail/breast-cancer)

4 The data is obtained from NCI [accessed 14 02 2023]. (Website https://www.cancer.gov/types/breast/patient/breast-treatment-pdq)

5 The data is obtained from ATTC [accessed 15 02 2023]. (Website https://www.atcc.org)
